# Carbohydrate Core–Shell Electrosprayed Microcapsules for Enhanced Oxidative Stability of Vitamin A Palmitate

**DOI:** 10.3390/pharmaceutics15112633

**Published:** 2023-11-16

**Authors:** Elnaz Z. Fallahasghari, Marie Højgaard Lynge, Emma Espholin Gudnason, Kristin Munkerup, Ana C. Mendes, Ioannis S. Chronakis

**Affiliations:** 1 DTU-Food, Research Group for Food Production Engineering, Laboratory of Nano-BioScience, Technical University of Denmark, Kemitorvet B202, 2800 Kgs. Lyngby, Denmarkemmaespg@gmail.com (E.E.G.); 2 BASF A/S, Malmparken 5, 2750 Ballerup, Denmark; kristin.munkerup@basf.com

**Keywords:** co-axial electrospray, micro-encapsulation, vitamin, oxidative stability

## Abstract

Vitamin A is an essential micronutrient that is readily oxidized. In this study, the encapsulation of vitamin A palmitate (AP) within a core–shell carbohydrate matrix by co-axial electrospray and its oxidative stability was evaluated. The electrosprayed core–shell microcapsules consisted of a shell of octenyl succinic anhydride (OSA) modified corn starch, maltose (Hi-Cap), and a core of ethyl cellulose–AP (average diameter of about 3.7 µm). The effect of different compounds (digestion-resistant maltodextrin, soy protein hydrolysate, casein protein hydrolysate, and lecithin) added to the base core–shell matrix formulation on the oxidative stability of AP was investigated. The oxidative stability of AP was evaluated using isothermal and non-isothermal differential scanning calorimetry (DSC), and Raman and Attenuated Total Reflectance–Fourier Transform Infrared (ATR-FTIR) spectroscopy methods. The core–shell carbohydrate matrix minimizes the amount of AP present at the microparticle surface, thus protecting AP from oxidation. Furthermore, the most effective oxidation protection was achieved when casein protein hydrolysate was added to the core of the microcapsule due to hydrophobic and hydrogen bond interactions with AP and by the resistant maltodextrin in the shell, which acted as a filler. The utilization of ethanol as a solvent for the dispersion of the core compounds increased the hydrophobicity of the hydrolyzed proteins and contributed to the enhancement of their antioxidant ability. Both the carbohydrate core–shell microcapsule prepared by co-axial electrospray and the addition of oxidation protection compounds enhance the oxidative stability of the encapsulated AP.

## 1. Introduction

**Figure 1:**
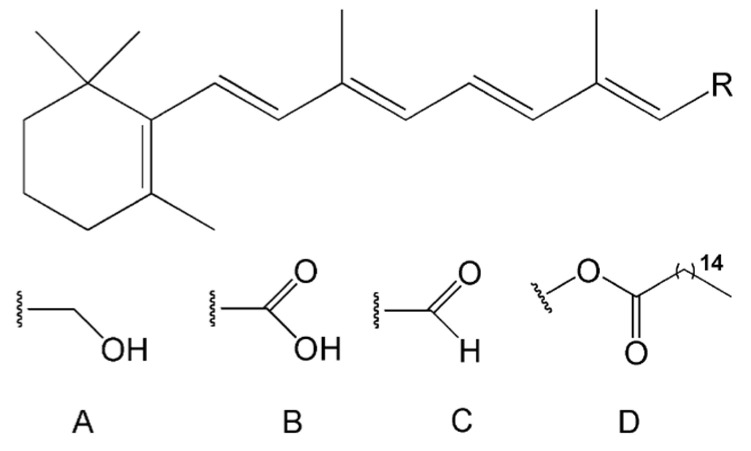
Selected retinoids structure where R can be: A, retinol; B, retinoic acid; C, retinaldehyde; D, vitamin A palmitate.

Vitamin A is a general term used for describing compounds with the qualitative biological activity of retinol, i.e., retinoids and some carotenoids (provitamin A) [1,2]. Vitamin A is an essential nutrient that plays an important role in the human body [3,4,5]. Vitamin A is well-known to be vital for preserving vision, supporting growth, and protecting the integrity of the body’s epithelium and mucosa. It also plays an important role in improving the immune system and enhancing the antibody reaction following many vaccinations [5,6]. Vitamin A deficiency, which leads to blindness and many other illnesses, is a public health problem, especially in developing countries [7]. Vitamin A-active retinoids are found in three basic forms: retinol, retinal, and retinoic acid (Figure 1) [3].

Retinoids are lipophilic compounds that are very sensitive to chemical degradation triggered by air (oxygen), light, heat, moisture, low pH, the presence of metallic ions, and oxidizing and reducing agents [8]. Among retinyl esters, vitamin A palmitate has higher stability than retinyl acetate, particularly when exposed to heat [9].

Electrohydrodynamic (EHD) atomization technologies (electrospray and electrospinning) have been studied for the encapsulation of bioactive compounds to protect or delay their degradation [10]. EHD processes utilize high-voltage electrostatic fields to charge the surface of (bio)polymer solution droplets and induce the ejection of a liquid jet through a spinneret. The EHD encapsulation processes can be performed at room temperature using a broad range of carbohydrates and proteins as an encapsulation matrix without compromising the functionality of sensitive bioactive compounds, including vitamins [10,11,12].

Taepaiboon et al. developed electrospun cellulose acetate nanofibers for the encapsulation of all-*trans* retinoic acid and α-tocopherol (vitamin E). Smooth fibers with an average diameter of 247–265 nm were obtained, and the release properties of the vitamins were investigated [13]. Lemma et al. reported a prolonged shelf life and higher thermal stability for the nanofibers of retinyl acetate produced with single nozzle electrospinning using polyvinyl alcohol (PVA) and β-cyclodextrin (β-CD). The structure of β-CD (with seven glucopyranose units and the inner cavities) enables the encapsulation of vitamin A and the formation of stable inclusion complexes [14]. Fahami et al. investigated the encapsulation of vitamin A within electrospun nanofibers of cress seed mucilage/polyvinyl alcohol. Change in the structure of nanofibers from amorphous to crystalline and an increase in the thermal stability of vitamin A was observed due to the hydrogen bond formation between vitamin A and cress seed mucilage/polyvinyl alcohol [15].

Other studies have been focused on the encapsulation of β-carotene, a provitamin of Vitamin A. For instance, Gómez-Mascaraque et al. encapsulated β-carotene in electrospray microparticles of protein matrices (zein and whey) using soybean oil as a lipid carrier and different emulsification procedures (high-speed homogenization and ultrasonication). Enhancement of the bioaccessibility of the carotenoids was observed, except for the microparticles prepared with whey protein using high-speed homogenization [16]. Mahalakshmi et al. also reported effective encapsulation of β-carotene by electrospray within zein and glycerol matrix with different core-to-shell ratios. The Fourier Transform Infrared (FTIR) results confirm hydrophobic interactions between the β-carotene and pyrrolidine ring of proline-rich zein protein and the binding of β-carotene with the functional group (N—H bending) of zein [17]. Niu et al. investigated the stability at different temperatures and relative humidity of β-carotene encapsulated within pullulan–whey protein isolate and medium-chain triglyceride capsules by electrospray [18]. Basar et al. also encapsulated β-carotene within whey protein capsules via emulsion by electrospray. The capsules showed excellent photo-oxidation stability (72% of β-carotene remained after 180 min exposure to UV light) [19]. 

In another study, β-carotene was encapsulated within whey protein nanocapsules by electrospray processing. Different concentrations of ethanol (5 to 15% *w*/*w*) were used for solubilization of β-carotene. It was observed that increasing the content of ethanol resulted in an increase in the size of the nanocapsules (from 227 to 283 nm). Interestingly, it was found that by increasing the ethanol content, an increase in the whey protein unfolding and aggregation occurred. The higher degree of the protein unfolding resulted in a higher exposure of its reactive groups, allowing the hydrophobic β-carotene to interact with the protein’s hydrophobic core and contribute to the protein structural rearrangements [20]. 

Furthermore, a variety of carbohydrates (e.g., cellulose derivatives, starch, and maltodextrin) have been used for the nano-microencapsulation of vitamin A and other lipophilic bioactive compounds [10]. For instance, Ma et al. and Liu et al. proposed an anti-solvent method for the development of ethyl cellulose particles loaded with α-tocopherol for inhibiting thermal oxidation of soybean oil. It was observed that by increasing the viscosity of the ethyl cellulose, the particle size was decreased, and the antioxidant activity was increased. This was due to the interactions of ethyl cellulose with α-tocopherol mainly via hydrogen bonds and hydrophobic interactions inhibiting the oxidation of soybean oil [21,22]. Differential scanning calorimetry and thermogravimetric analysis were used to study the degradation and oxidation of the particles [21]. 

Arayachukeat et al. encapsulated retinyl acetate by solvent replacement method using ethyl cellulose, poly (ethylene glycol)-4-methoxycinnamoylphthaloylchitosan (PCPLC) and studied the dermal penetration and release of retinyl acetate. It was found that ethyl cellulose improved the stability of the capsules in water; however, no improvement in the photostability of the vitamin was observed [23].

Moreover, octenyl succinic anhydride modified starches (OSA-modified starches) and maltodextrin has also been used as a carrier for the encapsulation of bioactive compounds due to their multifunctional properties, such as the binding ability to oil, bulk, and film formation and reduction in oxygen permeability [24,25,26]. OSA modification alters the hydrophilic character of the native starch to hydrophobic by substitution of octenyl molecules, resulting in an amphiphilic carbohydrate molecule [25]. OSA-modified starches are highly surface active and very effective in reducing surface–interfacial tension, and their emulsification properties are better than native starch due to both hydrophilic and hydrophobic characteristics [27]. Typically, OSA starches have low viscosities, which is extremely important for spray drying, extrusion, and other encapsulation processes [24].

A combination of maltodextrin and OSA-modified starch (Hi-Cap) has been used for the encapsulation of AP by spray drying. Starch formed a stable oil-in-water emulsion and enhanced the stability of AP [28]. Microencapsulation of vitamin A by spray drying using a mixture of gum arabic, maltodextrin, and starch and control release of the capsules (size of 6.94 to 11.61 μm) were also studied [29]. The release profile of vitamin A was influenced by the shell materials of the particles; maltodextrin provided a sustained release, while the ternary blend provided an intermediate release due to the contribution of the three encapsulating agents [29]. Moreover, in another study, vitamins A and E were encapsulated by emulsification and spray drying within maltodextrin–Capsul^®^ or maltodextrin/sodium caseinate particles for feed industry applications [30]. The porosity of the matrix is a crucial factor for the oxidative stability of the powders due to oxygen diffusion; porosity values above 70% might result in unwanted oxidation reactions. The thermal properties of the powders were also evaluated by DSC and thermo-oxidative decomposition measurements by thermogravimetric analysis (TGA) during storage time [30].

Co-axial electrospray processing permits the fabrication of capsules with a core–shell structure [10,31]. Only a few studies have assessed the co-axial electrospray processing for the encapsulation of lipophilic compounds such as essential oil, lycopene, α-linolenic acid, and fish oil [32]. In a recent study, the impact of mono-axial and co-axial encapsulation methods on the oxidative stability of fish oil was investigated. Low molecular weight carbohydrates (glucose syrup and maltodextrin) as the encapsulation matrix were used. Higher oxidative stability of the neat fish oil was observed for the capsules prepared using co-axial electrospray processing in comparison to mono-axial [33]. In the study by Drosou et al., β-carotene dissolved in corn oil and effectively encapsulated using two different matrices, pullulan (PUL) and its blend with whey protein isolate (WPI) (WPI:PUL 30:70 *w*/*w* blend), by co-axial electrospinning process. The WPI:PUL wall material provided greater protection of β-carotene against oxidative degradation under different storage temperatures, water activity levels, and upon exposure to UV–Vis irradiation than pure pullulan. It is suggested that this is probably due to stronger interactions between the protein components and the oil-β-carotene core, which minimizes the amount of β-carotene being present close to the surface of the nanofiber and thus making carotenoid molecules less amenable to oxidation by molecular oxygen [34]. 

Oxidation is the second most common degradation pathway for pharmaceuticals after hydrolysis. However, in contrast to hydrolysis, oxidation is mechanistically more complex, produces a wider range of degradation products, and is difficult to control and monitor [35]. The present study investigated the development of carbohydrate core–shell electrosprayed microcapsules and their potential to protect AP against oxidation. The core–shell microcapsules consisted of a shell of OSA-modified starch (Hi-Cap) and a core of ethyl cellulose–AP. Furthermore, the effect of the addition of oxidation protection compounds within the core or the shell of the microcapsules, such as digestion-resistant maltodextrin, soy protein hydrolysate, casein protein hydrolysate, and lecithin, was also evaluated. DSC and Raman spectroscopy based methods were exploited for the assessment of the oxidative stability of AP.

## 2. Materials and Methods

Vitamin A palmitate (retinyl palmitate) (1.7 Mio IU/G) (AP) was generously provided by BASF (BASF A/S Ballerup, Denmark). Hi-Cap^®^ 100 (OSA modified corn starch (E 1450) (with 50% maltose) (H) was obtained from Ingredion (Westchester, NY, USA). Ethyl cellulose 48.0–49.5% (*w*/*w*) ethoxyl basis in the form of coarse powder (46080-250G-F) (E); soy protein acid hydrolysate powder (S1674-500G) (S); HY-Case^®^ SF (Casein acid hydrolysate from bovine milk) (C9386-500G) (C); and Tween^®^ 20 (T20), a non-ionic surfactant that is soluble in ethanol), were purchased from Sigma-Aldrich Chemicals (St. Louis, MO, USA). Deoiled Soya lecithin powder, Lecico P 900 IP (Non-GMO) (L), was acquired from Lecico (Hamburg, Germany). Fibersol^®^–2 (F) was provided by ADM Bio Science & Technology (Tianjin, China). n-Hexadecane, 99% pure, was from ACROS Organic (Geel, Belgium) and was employed for the preparation of the dilution series. For the preparation of electrospray solutions, ethanol absolute ≥99.9% from VWR Life Science BDH (Rosny-sous-Bois, France) and Milli-Q^®^ water (Elix Technology Inside, Merck (Darmstadt, Germany)) were used.

### 2.1. Preparation of Solutions for Co-Axial Electrospray

The shell solution for the co-axial electrospray was prepared by dissolving Hi-Cap^®^ 100 (90% *w*/*v*) into milli-Q water at room temperature (20 °C) using a magnetic stirrer (VWR, Radnor, PA, USA) at 400 rpm for 1 h and kept overnight to remove the air bubbles. The core solution for the co-axial electrospray was prepared by dissolving ethyl cellulose (8% *w*/*v*) in ethanol (stirring of about 18–20 h). On the day of electrospray, AP (15% *v*/*v*) was mixed with Tween 20 (20% *w*/*w*), with respect to vitamin A content, and added to the core solution in the dark and at room temperature (stirring of about 30 min). Different compounds were also added to the shell or to the core solution, as shown in Table 1. Soy protein acid hydrolysate (2.5% *w*/*w*), casein acid hydrolysate (2.5% *w*/*w*), lecithin (2.5% *w*/*w*), or Fibersol (10% *w*/*w*) were added to the core formulation. Fibersol (20% *w*/*w*) was also added to the shell formulation. To avoid oxidation of AP, the electrospray process was performed immediately after the preparation of the solutions.

### 2.2. Co-Axial Electrospray Processing

Co-axial electrospray was performed in the closed system under nitrogen gas with a relative humidity of 30 ± 5% at room temperature. A lab-scale electrospray setup was used to develop core–shell structured microcapsules. To avoid the dripping of the solutions on the collector, the electrospray system was set horizontally. The feed rates of the core and shell solutions were controlled by two syringe pumps (New Era Pump System Inc., Farmingdale, NY, USA). The solutions were loaded into two 10 mL plastic syringes and delivered through silicone tubes to a three-fluid spray nozzle (Part of BUCHI B-290 Mini Spray Dryer B-290, BÜCHI Labortechnik AG, Essen, Germany) with an aligned inner needle with an interior diameter (I.D.) of 0.7 mm and outer needle with 2 mm I.D. Steady flow rates of 0.01 mL/min for the shell and 0.005 mL/min for the core solutions were applied. The system was equipped with a high-voltage power supply (Gamma High Voltage Research, Ormono Batch, FL, USA); 40 kV was applied between the nozzle tip and the grounded collector. A stainless-steel plate (30 cm × 30 cm) covered with aluminum foil was used as a collector, and the distance between the needle tip and the collector was 30 cm.

### 2.3. Morphological Characterization of the Microcapsules by Scanning Electron Microscopy (SEM)

The Morphological characteristics of microcapsules were investigated by SEM using a Quanta FEG 200 Cyro ESEM (Environmental Scanning Electron Microscope) (FEI Company, Hillsboro, OR, USA) at the accelerating voltage of 10 kV, a working distance of about 10 mm, and a magnification of 3000× and 5000× by a secondary electron detector (Everhart–Thornley detector—ETD). Electrospray microcapsules on the aluminum foil (approximately 0.5 cm × 0.5 cm) were transferred and attached to SEM sample holders with carbon adhesive tapes (agar scientific). To reduce the sample charge and to have a better resolution, the samples were gold sputter-coated for 20 s and a sputtering current of 20 milliamperes (mA), using an automatic sputter coater, Q 150T (Quorum Technologies Ltd., Lewes, UK), prior SEM imaging.

The size distribution of the microcapsules was investigated by utilizing ImageJ 1.47t (National Institute of Health, Bethesda, MD, USA). For each sample, the size distribution curve was obtained by randomly measuring one hundred capsules’ size per image using OriginPro 2021 9.8.0.200 (OriginLab Corporation, Northampton, MA, USA). 

### 2.4. Evaluation of the Oxidative Stability of the Microcapsules

#### 2.4.1. Evaluation of the Oxidative Stability by Differential Scanning Calorimetry (DSC)

The oxidative stability of the microcapsules was determined by both isothermal and non-isothermal DSC methods using a DSC 250 (TA Instruments Ltd., New Castle, UK) according to [36] with some modifications. The DSC was calibrated with high-purity indium standard, and the data were measured when the gas switched from nitrogen to oxygen (99.995% purity) with the maintained flow of 50 mL/min. In both methods, 5–5.9 mg (in order to retain the same content of vitamin A for all the samples) were weighed into an open aluminum pan and placed in the DSC instrument tray. An empty aluminum pan with an open lid was used as a reference.

In the isothermal DSC method, the oxidative induction time (OIT) procedure was adapted from Micić et al. [36]. In the first stage, the sample and reference were kept at 10 °C for 5 min and then heated to 140 °C with a heating rate of 5 °C/min under the nitrogen atmosphere. After holding the DSC cell for 5 min isothermally, the pans were subjected to oxygen gas for 120 min. The area under the isotherm OIT procedure curve was used to quantify and compare the oxidation properties of the microcapsules. A calibration curve of non-encapsulated AP (in the range of 1–11 mg) was used to compare the oxidative stability of non-encapsulated AP with the encapsulated AP samples. 

The non-isothermal DSC method, oxidative onset temperature (OOT), was carried out according to the method used by N. Vilanova and C. Solans [37] with some modifications. The sample and the reference pans were equilibrated at 30 °C for 5 min under a nitrogen atmosphere. Then, the gas injection was switched to oxygen, and the sample was heated to 300 °C with a heating ramp of 5 °C/min. The non-isothermal DSC curve exhibited two peaks; typically, the first peak is considered the first stage of oxidation [38]. The enthalpy of the first peak on the non-isothermal DSC curve of the encapsulated and non-encapsulated AP samples was evaluated. The non-isothermal and isothermal measurements of each sample were carried out in duplicate.

#### 2.4.2. Evaluation of the Oxidative Stability of AP by Raman Spectroscopy

**Figure 2:**
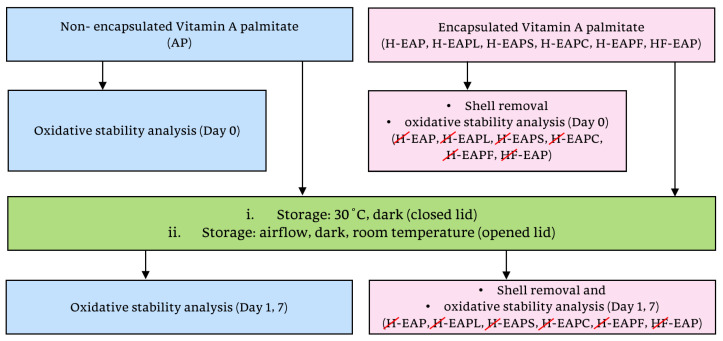
Experimental design for the oxidative stability of non-encapsulated AP and microcapsules with AP under different storage conditions.

A storage experiment with two severe oxidative conditions for AP was performed (Figure 2): (i) incubation of the samples at 30 °C, darkness (within tubes with a closed lid); (ii) under airflow, darkness at room temperature (within Petri dishes (D: 50 mm) with an open lid).

In order to evaluate the oxidation of AP by spectroscopical methods, it was necessary to remove the carbohydrate shell (Hi-Cap) of the microcapsules. For the removal of the shell, about 50 mg of the microcapsules were suspended in 1 mL of water and vortexed (IKA VF2, IKA Labortechnik, Staufen, Germany) for 1 min in an Eppendorf tube. After centrifuging (using an Eppendorf Centrifuge 5424, Hamburg, Germany) at 1400 rpm for 10 min, the supernatant was carefully discarded, and the spectra of the pellet fraction were measured both with Raman microscopy. 

The Raman spectroscopy was conducted using the DXR3 Raman microscope (Thermo Fischer Scientific, Waltham, MA, USA) equipped with Omnic 9.12.928 software. All the acquisitions were performed with a laser wavelength of 532 nm, laser power of 8 mW, a 10× magnification, and a 50-pinhole (in non-encapsulated AP 25-pinhole) aperture within the range of 4000 to 400 cm^−1^. For the collection of a representative sample, different sample positions (10 points) inside an area map (mosaic) were chosen randomly. Baseline corrections were also performed. 

#### 2.4.3. Evaluation of the Interactions of AP with the Core Compounds of the Microcapsule by ATR-FTIR Spectroscopy

For the determination of the possible reactions of AP with the core compounds of the microcapsules, FTIR analysis of the core solutions was performed after evaporation of the solvent (evaporation of the ethanol using nitrogen gas in darkness was necessary to obtain better resolution of the FTIR data). The FTIR spectra were recorded by utilizing a Nicolet iS50 FT-IR spectrometer (Thermo Fisher Scientific, Waltham, MA, USA) equipped with a built-in ATR sampling device. All the spectra were acquired at room temperature within the wavenumber of 4000–600 cm^−1^ with an aperture of 230. Each spectrum was collected with a resolution of 4 cm^−1^ in the absorbance mode and represented an average of 32 scans. 

### 2.5. Encapsulation Efficiency

The encapsulation efficiency (EE) of the microcapsules was assessed according to [39] with some modifications. This method is based on the removal of non-encapsulated material located on the surface of the capsules with a proper solvent. An amount of 25 mg of the electrosprayed capsules were submerged in hexadecane (5 mL) and gently shaken with IKA MS 3 digital shaker ((IKA^®^—Works Inc., Wilmington, NC, USA) at 100 rpm for 15 min. Then, the suspension was filtered through quantitative grade 1 filter paper (11 µm particle retention) (Whatman, Buckinghamshire, UK). The absorbance of the liquid was measured at 362 nm using a NanoDrop^TM^ One/One^C^ Microvolume UV-Vis Spectrophotometer (Thermo Fisher Scientific, Madison, WI, USA). A calibration curve with a serial dilution of AP as standard and hexadecane as solvent was prepared. The amount of non-encapsulated AP in the hexadecane, derived from the surface of microcapsules, was determined by using the calibration curve (R^2^ = 0.99). The *EE* value was measured as follows:

$$EE\left(\%\right)=\frac{A-B}{A}\;*\;100$$

where *A* is the theoretical or initial amount of AP in the formulation of the microcapsules, and *B* is the amount of free AP that is collected in hexadecane. The measurements were carried out in triplicate. 

### 2.6. Statistical Analysis

For statistical analysis, Statgraphics Centurion, version 18 (Statistical Graphic Crop., Rockville, MD, USA), was used. The mean, standard deviation, and standard error were calculated. *t*-test and ANOVA were used for comparison of the mean values and the determination of the significant differences between samples. The criteria for stating whether the result was significant was the *p*-value. The number of asterisks used in each graph indicates the level of significance. One asterisk (*) indicates a *p*-value equal to or less than 0.05, two asterisks (**) are used for a *p*-value equal to or below 0.01, and three asterisks (***) signify the *p*-value is equal to or less than 0.001. 

## 3. Results and Discussion

### 3.1. Morphology of the Electrospray Core–Shell Microcapsules

**Figure 3:**
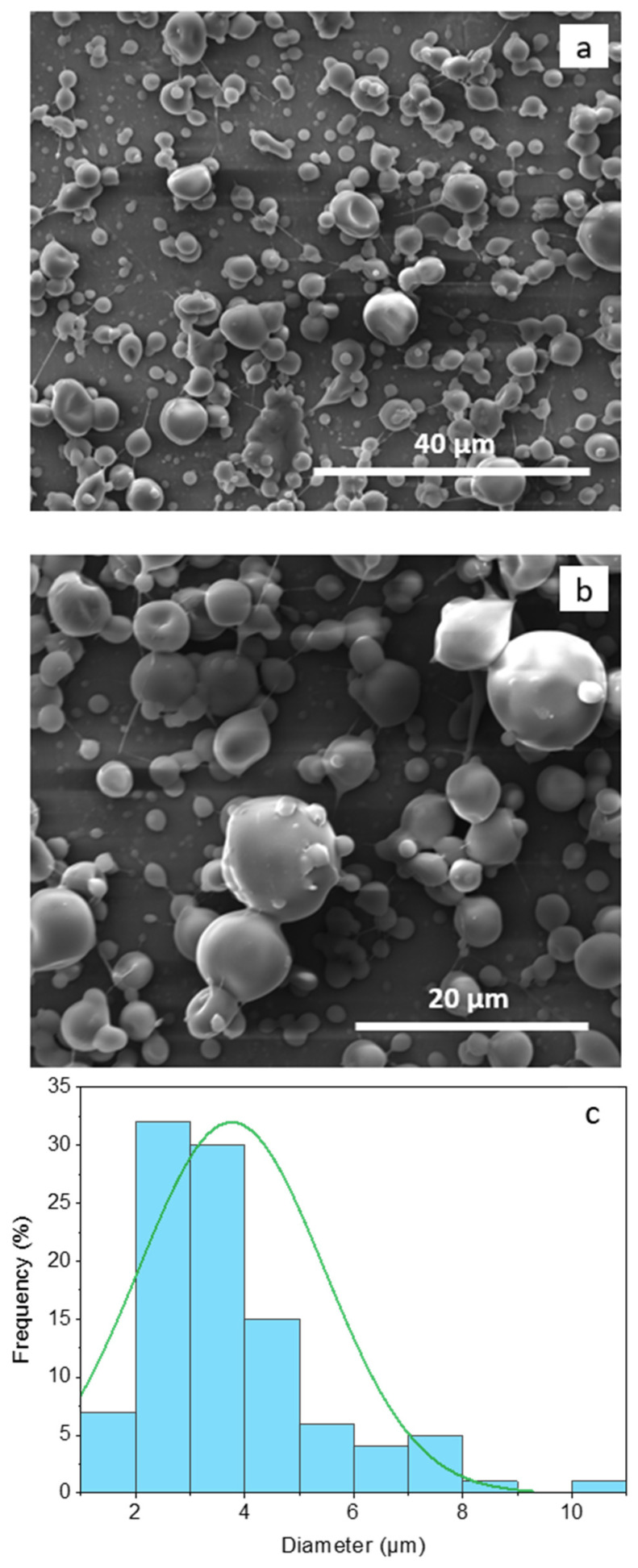
SEM images of H-EAP core–shell microcapsules at different magnifications (**a**,**b**) and corresponding histogram displaying their diameter distribution (**c**).

The size and the morphology of electrospray core–shell microcapsules loaded with AP (H-EAP) are shown in Figure 3. The core–shell microcapsules showed a spherical structure with a smooth spherical surface, uniform morphology, and an average diameter of about 3.7 ± 1.7 µm. The formation of some concavities on the surface of the microcapsules could be observed due to the fast evaporation of the highly volatile ethanol solvent from the core during the electrospray process [40]. Typically, electrospray capsules with smoother surfaces can be obtained using solvents with lower evaporation rates [10,31]. It is to be noted that the low viscosity of Hi-Cap starch permits the preparation and the electrospray of a shell carbohydrate matrix at higher solids (90% *w*/*v*); this formulation can be electrospray-dried at faster rates, as less water has to be evaporated. 

The morphology and the average diameter of the electrosprayed capsules with the addition of the oxidation protection compounds (Fibersol-2, lecithin, soy protein hydrolysate, casein hydrolysate in the core or Fibersol-2 in the shell, Table 1) are similar to the morphology of the H-EAP capsules. Moreover, no morphological changes were observed for the microcapsules stored for up to seven days under the different oxidation conditions of airflow and temperature of 30 °C. The encapsulation efficiency of AP within the microcapsules is high (in the range of 98.20 to 98.65% for the capsules shown in Table 1). The high EE can be attributed to the interactions between AP with the dispersed ethyl cellulose–core compounds, which lead to high entrapment of AP, as well as due to the core–shell structure and the efficiency of the electrospray processing [11,40].

### 3.2. Assessment of the Interactions of Vitamin A Palmitate with the Core Compounds of the Microcapsules by FTIR Spectroscopy

**Figure 4:**
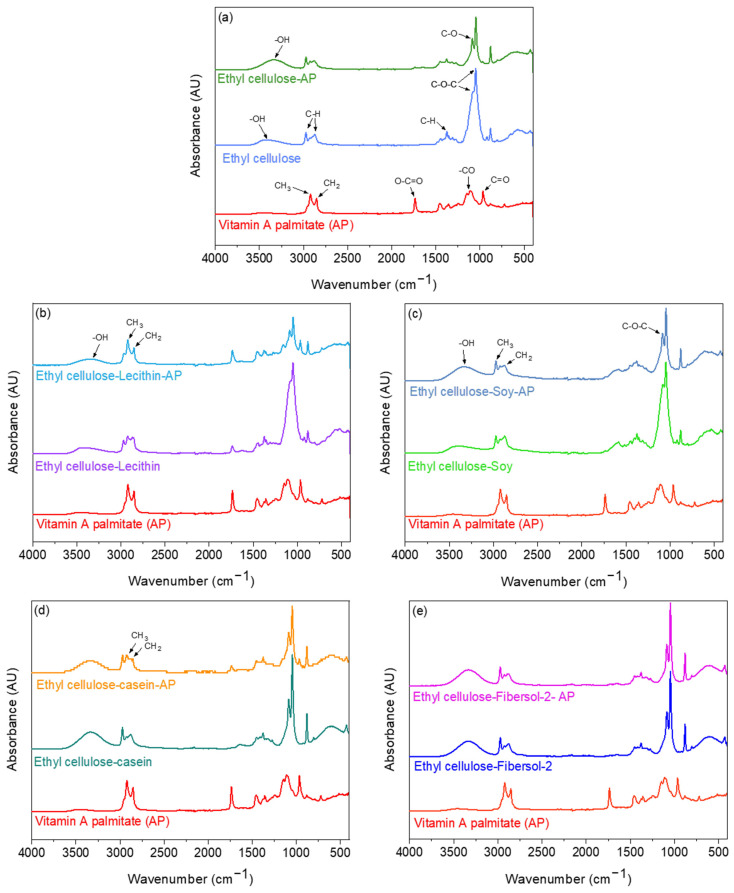
FTIR spectra of vitamin A palmitate (AP) with the core compounds of the microcapsules (after suspension in ethanol and evaporation of the solvent). (**a**) AP, ethyl cellulose, and ethyl cellulose–AP; (**b**) AP, ethyl cellulose–lecithin and ethyl cellulose–lecithin–AP; (**c**) AP, ethyl cellulose–soy protein hydrolysate, ethyl cellulose–soy protein hydrolysate–AP; (**d**) AP, ethyl cellulose–casein protein hydrolysate, ethyl cellulose–casein protein hydrolysate–AP; (**e**) AP, ethyl cellulose–resistant maltodextrin (Fibersol-2), ethyl cellulose–Fibersol-2–AP.

The FTIR spectra of AP (after suspension in ethanol and evaporation of the solvent) (Figure 4a) showed different characteristics bands of AP, which include C-H bonds stretching of CH3 and CH2 groups at 2921 and 2852 cm^−1^, respectively [30,41]. The peak at 1736 cm^−1^ is attributed to (O–C=O) stretching from the ester functional groups [30,41]. The peaks in the region between 1460 cm^−1^ and 1160 cm^−1^ are assigned to the stretching vibration of –CO groups [30]. In this region, peaks at 1456, 1358, and 1160 cm^−1^ were measured. The peak in the region of 1100 cm^−1^ was assigned to carbonyl stretching [41].

The FTIR of ethyl cellulose (after suspension in ethanol and evaporation of the solvent) (Figure 4a) showed the main band at 3350 cm^−1^ due to the –OH groups of the polysaccharide backbone [42]. The asymmetric peaks of CH stretching can be seen in the peak regions of 2970 and 2870 cm^−1^. Other relevant vibration peaks around 1054, 1085, and 1375 cm^−1^ were mainly attributed to C-O–C stretch and C–H bending, respectively [43]. 

The FTIR of the ethyl cellulose–AP mixture (after suspension in ethanol and evaporation of the solvent) showed a shift at 3342 cm^−1^ and an increase in –OH band intensity as a result of hydrogen bond formation (Figure 4a). The methyl groups slightly changed comparatively to ethyl cellulose, suggesting that there are hydrophobic interactions between ethyl cellulose and AP. However, the spectra of the ethyl cellulose–AP mixture showed a narrower C-O-C stretch absorption band in comparison to the ethyl cellulose spectra, which might be related to changes in the bond strength [40]. Furthermore, an intense band at 1085 cm^−1^ appeared, which could be related to the C-O stretch of an alcohol group (retinol) and subsequent C-O-C changes in the glycosidic linkage due to changes in the conformation of the polymers [44].

FTIR spectroscopy was also used to investigate potential interactions between AP and core compounds of the microcapsule (Figure 4b–e). The FTIR spectra of the lecithin–ethyl cellulose–AP (after suspension in ethanol and evaporation of the solvent) showed shifts in the methylene groups at 2921 and 2852 cm^−1^, suggesting hydrophobic interactions. Furthermore, the –OH band also shifted, probably due to hydrogen bond formation between lecithin, ethyl cellulose, and AP. In the case of the spectra of casein protein hydrolysate with ethyl cellulose–AP mixture (after suspension in ethanol and evaporation of the solvent), major shifts in methylene groups at 2921 and 2852 cm^−1^ were also observed due to hydrophobic interactions. These shifts are higher in magnitude than the shifts found for the lecithin–AP–ethyl cellulose mixture, suggesting stronger interactions in the presence of casein hydrolysate. The spectra of soy protein hydrolysate with ethyl cellulose–AP (after suspension in ethanol and evaporation of the solvent) showed an increase in the –OH band intensity, in comparison to the ethyl cellulose–AP spectra, because of hydrogen bond formation. Furthermore, the methylene groups did not change comparatively to ethyl cellulose spectra, suggesting the absence of hydrophobic interactions between the soy hydrolysate and ethyl cellulose and AP. However, the band at 1088 cm^−1^, corresponding to C-O–C stretch, is more pronounced for the soy–ethyl cellulose–AP mixture, in comparison to soy–ethyl cellulose spectra, suggesting that the soy hydrolysate influenced this band. Finally, the FTIR spectra of resistant maltodextrin (Fibersol-2) with ethyl cellulose–AP showed the same vibrational bands and no major shifts in comparison to the resistant maltodextrin-ethyl cellulose spectra, suggesting that there are no interactions of the resistant maltodextrin with AP.

### 3.3. Oxidation Assessment of Non-Encapsulated and Encapsulated Vitamin A Palmitate by Raman Spectroscopy: Effect of Airflow and Temperature

**Figure 5:**
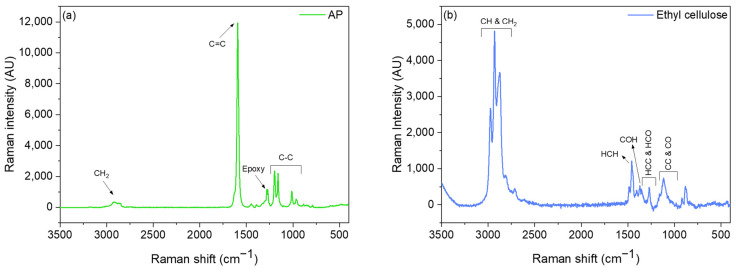
Raman spectra of (**a**) non-encapsulated vitamin A palmitate (AP) and (**b**) ethyl cellulose powder.

The Raman spectra of AP consist of a broad peak around 2900 cm^−1^, which corresponds to the methylene group of AP (Figure 5a). However, at 1280 and 1270 cm^−1^, two weak peaks were present that fit in the range of an epoxy group, and this could indicate the 5,6-Epoxy-AP [45,46,47]. These peaks also appeared in solid AP; thus, they are not due to the handling of AP. A main peak for AP appeared at 1593 cm^−1^, which is assigned to conjugated C=C stretching vibration of retinol [48,49], one of the retinyl palmitate derivatives, and confirms the FTIR band appeared at 1085 cm^−1^ related to the C-O stretch of an alcohol group. The double peaks seen at 1197, 1160, and 1012 cm^−1^ are related to C-C bonds [50].

The Raman spectra of the ethyl cellulose showed intense bands between 2800 and 3000 cm^−1^, attributed to CH and CH_2_ stretching vibrations (Figure 5b). Between 1430 and 1500 cm^−1^, the major bands can be related to HCH bending; from 1430 to 1350 cm^−1^, the major bands are related to COH bending; and from 1350 to 1270 cm^−1^, the bands are related to HCC and HCO bending. In the region between 950 and 1180 cm^−1^, several closely spaced bands are observed related to CC and CO stretching motions. The bands between 550 and 750 cm^−1^ are assigned to CCC, COC, OCO, CCO, and OH out-of-plane bending [51]. The OH bending modes are observed in infrared spectra but are absent from the Raman spectra because of the large dipole moment and low polarizability associated with the OH bond [51]. 

**Figure 6:**
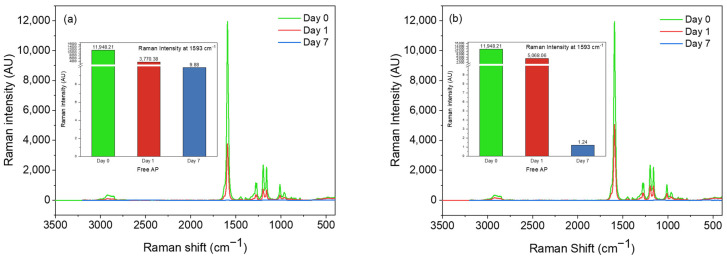
Raman spectra of non-encapsulated vitamin A palmitate (AP) incubated for 7 days: (**a**) at 30 °C and (**b**) under airflow.

Raman spectroscopy was also used to assess the oxidative stability of AP by exposing the non-encapsulated AP under airflow and 30 °C temperature. Figure 6a,b shows a substantial decrease in the intensity of the main peak assigned to C=C stretching vibration at 1593 cm^−1^ of non-encapsulated AP due to oxidation by airflow and temperature. The intensity of the peak decreased by 58% and 68% from day 0 to day 1 when non-encapsulated AP was oxidized by air and temperature, respectively. After seven days, the intensity of the peak at 1593 cm^−1^ decreased by nearly 100%. Overall, the Raman data confirm that the airflow had a higher effect on the oxidation of AP than the temperature (note that the non-encapsulated AP was stored at 30 °C in tubes with a closed lid).

**Figure 7:**
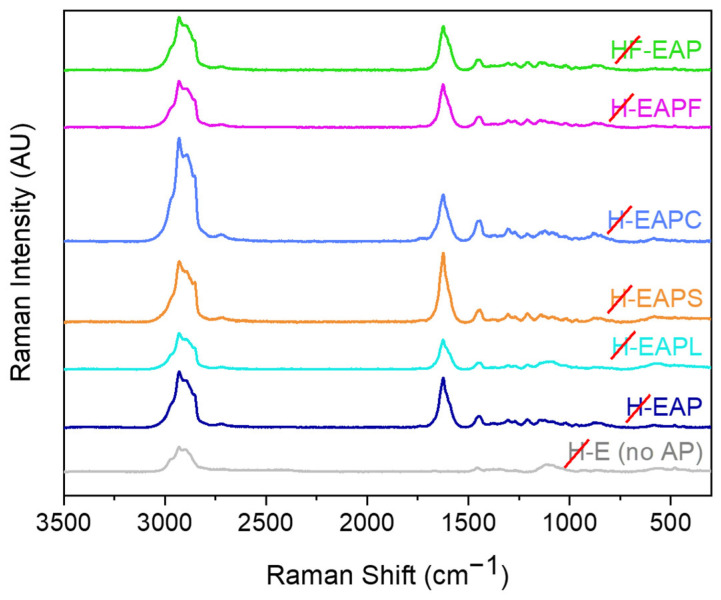
Raman spectra of encapsulated vitamin A palmitate (AP) and capsules containing no AP at day 0 (Raman spectra recorded after removal of the microcapsules shell).

**Figure 8:**
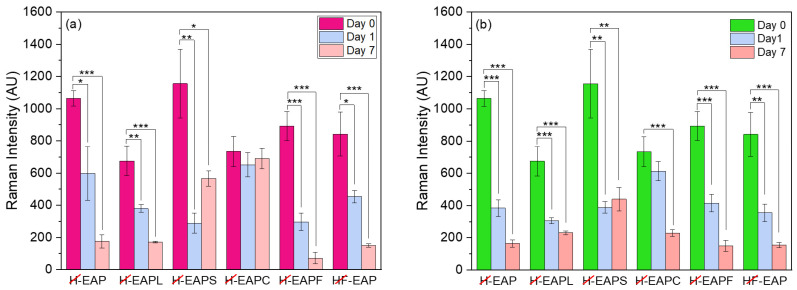
Raman intensity changes at 1593 cm^−1^ for the microcapsules with AP after storage for 7 days: (**a**) incubation at 3 + 0 °C (in tubes with a closed lid); (**b**) under airflow (Raman spectra recorded after removal of the microcapsules shell.) One, two, and three asterisks indicate the significant difference between different days in one sample with *p*-values of ≤0.05, ≤ 0.01, and ≤0.001, respectively.

The oxidative stability over time under the effect of airflow and temperature of 30 °C of the encapsulated AP within the carbohydrate core–shell matrix and the effect of the addition of oxidation protection compounds to this matrix was also evaluated by Raman spectroscopy. Figure 7 shows the Raman spectra of encapsulated AP (after shell removal) at day 0. A decrease in the peak intensity at 1593 cm^−1^ (corresponding to conjugated C=C) was observed for the microcapsules with AP stored either in the air or at 30 °C after seven days (Figure 8a,b). Clearly, the formulations with the casein protein hydrolysate in the core or the resistance maltodextrin in the shell showed the highest oxidative stability (Figure 8a,b).

### 3.4. Oxidation Assessment of Non-Encapsulated and Encapsulated Vitamin A Palmitate Using DSC

**Figure 9:**
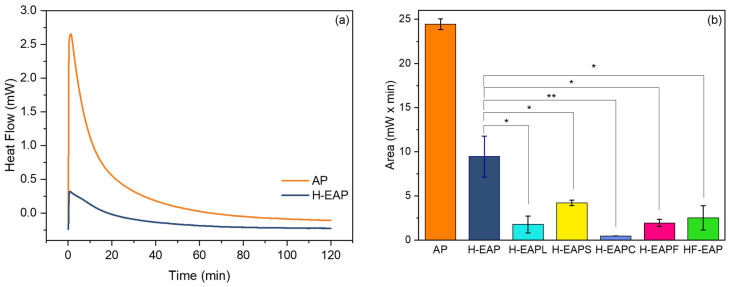
Isothermal DSC results. (**a**) Isothermal DSC oxidation curves of AP and HEAP at 140 °C and oxygen flow of 50 mL/min. (**b**) Area under the isothermal oxidation curve for non-encapsulated AP and microcapsules with AP. One or two asterisks indicate a significant difference between different encapsulated samples with *p* values of ≤0.05 or ≤0.01, respectively (**b**).

Figure 9a shows the isothermal DSC oxidation curves for the AP and AP encapsulated within the Hi-Cap-ethyl cellulose core–shell microcapsule (H-EAP). A large exothermic peak was clearly shown for the non-encapsulated AP, which is considerably decreased for the encapsulated vitamin A. The area under the isothermal curve was used to quantify and compare the oxidative stability of the microcapsules (Figure 9b). The area under the isothermal curve for non-encapsulated AP was nearly three-fold higher (24.44 mW × min) in comparison to the area of the H-EAP sample (9.47 mW × min), indicating the high oxidative stability that is provided by the carbohydrates matrix of Hi-Cap and ethyl cellulose. Thus, encapsulation of AP by co-axial electrospray prevented the presence of vitamin at the surface of the microcapsule and protected it from oxidation. Additionally, the dense carbohydrate matrix probably increased the oxidative stability of AP by enhancing the impermeability to oxygen and by providing thermal stability.

The addition of the oxidation-protecting compounds in the core or shell of the microcapsule (Table 1) further promoted the oxidative stability of AP (Figure 9b). The thermal stability of all the encapsulated AP samples indicates an effective encapsulation against degradation and oxidation. Particularly, for the compounds added to the core, casein hydrolysate was the most protective compound (H-EAPC, area of 0.495 mW × min), whereas soy protein hydrolysate was the least protective one (area of 4.23 mW × min). It has been suggested that the use of protein hydrolysates favors the exposure of hydrophobic groups and amino acid residues capable of inhibiting lipid oxidation through different mechanisms of action (e.g., radical scavenging and/or metal chelation) [52,53,54]. Additionally, small peptides produced as a result of protein hydrolysis could also act as a ‘filler’ of the encapsulating wall [54,55]. In the above studies, however, the protein hydrolysates were dispersed in an aqueous phase rather than pure ethanol, as in the current study.

The addition of the digestion-resistant maltodextrin (Fibersol-2) in the core or shell of the microcapsule also further protects from the oxidation of AP (area of 1.95 mW × min and 2.53 mW × min, respectively), supporting that the addition of the carbohydrate acted as ‘filler’, most probably limited the oxygen diffusion, prevented the diffusion of AP to the surface, and provided resistance to oxidative degradation of AP. Note that the concentration of the resistant maltodextrin was much higher in comparison to the other compounds. The carbohydrate has a low viscosity, and it was possible to disperse it at higher concentrations both at the core or at the shell formulations. 

**Figure 10:**
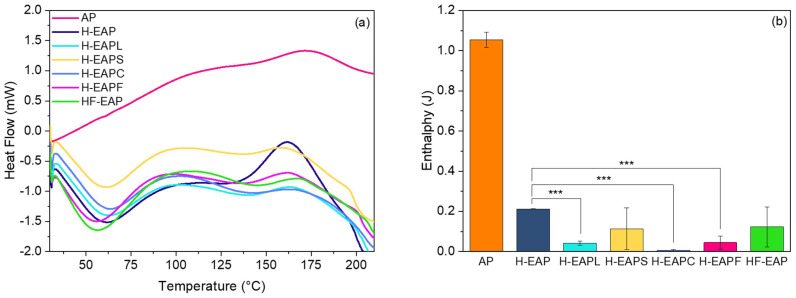
Non-isothermal DSC results: (**a**) DSC curves during heating of non-encapsulated AP and microcapsules with AP. (**b**) Enthalpy obtained from the first oxidation peak of the non-isothermal DSC curves for the non-encapsulated AP and for the microcapsules with AP. Three asterisks indicate a significant difference between samples with a *p* value of ≤0.001.

Moreover, from the non-isothermal measurements (Figure 10a) and the evaluation of the enthalpy (Figure 10b), it was also observed that the casein hydrolysate (in the core of the microcapsule) contributed the highest to the oxidative stability of AP (the enthalpy of H-EAPC was hundred times lower than the non-encapsulated AP), and thus significantly delayed the degradation of AP. The addition of other oxidation protection compounds to the core also showed low enthalpy values. It is to be noted that the enthalpy of the H-EAP microcapsule is nearly five times lower than the non-encapsulated AP, further confirming that the dense carbohydrate matrix increased the oxidative stability of AP. Overall, the DSC data are in agreement with the Raman and FTIR results, supporting that both the carbohydrate core–shell matrix and the inclusion of the various compounds at the core or at the shell of the microcapsule improved the oxidative stability of AP.

To the best of our knowledge, the area under the oxidation curve in the isothermal method has not been used to evaluate the oxidative stability of lipophilic compounds and vitamin A. The area under the DSC curve for the non-isothermal method has been used to study the thermal degradation of compounds such as biomasses [56]; however, it has not been utilized to compare the oxidation of bioactive compounds. In a study by Micić et al., both isothermal and non-isothermal DSC methods were used to evaluate the oxidative stability of blackberry and raspberry seed oil under the effect of oxygen [36]. Regarding the non-isothermal DSC method, the oils were heated at different heating rates, and the slope of the onset temperature (heat flow versus temperature) was used to evaluate the oxidation process. In the isothermal method, the process of oil oxidation appeared as an exothermic heat flow, and the oxidation induction time (OIT) for each oil was evaluated from the intersection of the extrapolated baseline and tangent line of the exothermic peak [36]. In another study, Vilanova and Solans also investigated the protecting ability of water-soluble inclusion complexes of vitamin A palmitate with β-cyclodextrins, using DSC under oxidative conditions in the OOT method. An exothermic peak for free vitamin A palmitate was observed, whereas an endothermic curve was observed for the inclusion complex, suggesting oxidative stability of the immobilized vitamin within the β-cyclodextrin cavity [37]. Other studies have used the glass transition temperature, the melting temperature, and the melting enthalpy from the DSC measurements to assess the stability of the vitamin A encapsulated within spray-dried Hi-Cap microcapsules [57].

## 4. Further Discussion on the Enhanced Oxidative Stability of the Encapsulated Vitamin A Palmitate

Ethanol has a dissociative impact on proteins and peptides; it disrupts their hydrophobic interactions or hydrogen bonds, induces the unfolding of proteins, and increases protein hydrophobicity [58,59]. In the present study, soy protein hydrolysate and casein protein hydrolysate are dispersed in ethanol within the ethyl cellulose–AP solution. These protein structures most probably unfold in ethanol, leading to the exposure of their hydrophobic amino acids and the formation of clusters incorporating the AP. Immobilization of vitamin A by binding to these aggregates and interactions with the protein’s hydrophobic core decreased the vitamin’s mobility and chemical reactivity, and acted as a physical barrier against oxidizing agents. 

Previous studies have also confirmed that moderate ethanol pretreatment (up to 40%) increased the antioxidant activities of WPI through molecular unfolding of the native protein [60,61,62]. Specifically, ethanol pretreatment increased the exposure of aromatic amino acids and sulfur-containing amino acids of WPI (such as cysteine, methionine, tyrosine, and phenylalanine) that typically contribute to the antioxidant ability of proteins [63]. Moreover, it was found that the content of hydrophobic amino acids (such as alanine, phenylalanine, isoleucine, glycine, leucine, valine, proline, and methionine) in ethanol-treated WPI samples also increased. It was concluded that the high content and hydrophobicity of these amino acids might contribute to the high antioxidant ability of the ethanol-treated WPI. Similarly, the addition of the lipophilic vitamin D dissolved in ethanol to caseinate water solution initiated the vitamin’s self-aggregation and, subsequently, adsorption of the casein onto the vitamin D aggregates; thus, the formation of stable nanosized co-assemblies enhanced the stability of vitamin D [64]. Casein also has intrinsic stability to form protein–ligand complexes, and therefore casein micelles have been exploited for the encapsulation of lipophilic vitamins. The structure and the amphiphilic properties of caseins cause a rapid adhesion of the protein to the droplet surface of the lipophilic compounds, forming a thick and entangled protecting layer [30,64]. It has also been suggested that caseins have anti-oxidative and free radical quenching capabilities due to other free thiol groups of kappa-casein [65].

Liu et al., 2019, also utilized ethanol for disassembly–reassembly and fabricated a kind of soy β-conglycinin (β-CG; a major storage globulin from soybeans) nanoparticles that were used as highly efficient nanocarriers for the encapsulation of hydrophobic nutraceuticals (using curcumin as a model bioactive) [59]. This study also confirmed that when the β-CG was treated with ethanol at high concentrations (e.g., >30%, *v*/*v*), its structure gradually unfolded, and protein denaturation and aggregation occurred, while the reassembled β-CG particles (at ethanol of 40%) exhibited the highest capacity to load curcumin, and much greater stability and bioaccessibility than free curcumin. Furthermore, due to the formation of hydrogen bonds, the thermal stability of vitamin A encapsulated within electrospun cress seed mucilage/polyvinyl alcohol nanofibers has been enhanced [15]. Both hydrogen bonds and hydrophobic interactions of ethyl cellulose with α-tocopherol have also been found to inhibit the oxidation of soybean oil [21,22]. Moreover, soy peptide nanoparticles by self-assembly of large peptide aggregates, which are induced by ultrasonication in an oil-in-water emulsion, showed a great suppression in the oxidation of corn oil both in lipid hydroperoxide value and volatile hexanal [66]. Other studies further support the high antioxidant activities of soy protein isolate hydrolysates in aqueous and liposomal systems [67,68].

Furthermore, lecithin is a known antioxidant and typically reduces the permeation of free radicals across the emulsion interface [69,70], as well as prevents oxidation by chelating metals [71]. In this study, the oxidation protective effect of lecithin is probably due to the hydrophobic interactions and hydrogen bond formation between lecithin, ethyl cellulose, and AP. Pezeshky et al. also studied the effect of different concentrations of lecithin–cholesterol for the encapsulation of vitamin A palmitate within nanoliposomes using thin-film hydration–sonication method. A complex formation between vitamin A and liposomes by physical interactions was found, while an association between lecithin concentration, entrapment efficiency of vitamin A palmitate, and the loading capacity of nanoliposomes was also observed [41].

Fibersol-2 is a prebiotic dietary fiber-resistant maltodextrin, partially hydrolyzed by human digestive enzymes (dextrose equivalent of 8–12.5, average molecular weight of 2000 Da). It is composed not only of α(1→4) and α(1→6) glucosidic bonds, as are present in the native starch, but also contains 1→2 and 1→3 linkages and levoglucosan [72]. Due to the high ratio of 1–6 glucosidic bonds-branched structure, it is suitable for stabilizing micellar emulsions [73]. Particularly, it was found that Fibersol-2 inhibited the decomposition of micelles and stabilized micellar structure (even in the digestive tract) by suppressing lipid absorption and promoting the excretion of lipids into feces by delaying the release of fatty acids from the micelles in the lipid absorption process. Thus, these structural characteristics of the resistant maltodextrin could contribute to the stabilization of the electrospray core dispersion of the lipophilic AP. Moreover, the low molecular weight of the Fibersol-2, its branched structure, and its low hygroscopicity may have also contributed to the formation of a more densely packed electrospray shell matrix (when Fibersol-2 added as an extra carbohydrate compound at the shell layer), provided effective barrier properties that limited the diffusion of oxygen and humidity, and enhanced the oxidative stability of AP. Note that due to the low viscosity and high water solubility of Fibersol-2, it has been dispersed to 20% *w*/*v* within the Hi-Cap shell carbohydrate matrix and up to 10% within the core. Few other studies have also assessed the utilization of resistant maltodextrin as an encapsulating shell material for bioactive compounds using, however, spray-drying and freeze-drying processing [57,74,75,76].

## 5. Conclusions

The encapsulation of vitamin A palmitate within a core–shell carbohydrate matrix by co-axial electrospray and its oxidation stability was evaluated. Overall, the core–shell matrix minimizes the amount of AP being present at the microparticle surface, thus protecting AP from oxidation. The FTIR data of AP with the core compounds of the microcapsules support that casein protein hydrolysate, which is suspended in ethyl cellulose–AP ethanol dispersion, most probably unfold, leading to the exposure of its hydrophobic amino acids and forms aggregated clusters that incorporate the AP. The hydrophobic interactions of AP with the protein’s hydrophobic core decreased the vitamin’s mobility and chemical reactivity, as well as the immobilization of vitamin A at protein aggregates, which may act as a physical barrier against the penetration of oxidizing agents. It was also found that lecithin in the core of the microcapsule is effective in protecting AP from oxidation, probably due to the hydrophobic interactions and hydrogen bond formation between lecithin, ethyl cellulose, and AP. The hydrogen bond formation between ethyl cellulose and AP with the unfolded soy protein hydrolysate structure in ethanol mainly contributes to the increased oxidative stability of the vitamin. Furthermore, digestion-resistant maltodextrin, both in the shell or at the core of the microcapsule, acted as a filler, provided effective barrier properties, and enhanced the oxidative stability of AP. This work highlights the importance of utilizing and functionalizing core–shell microcapsules to enhance the oxidative stability of lipophilic bioactive compounds for nutritional and pharmaceutical applications. 

## Figures and Tables

**Table 1:** Formulations used for the preparation of core–shell microcapsules by co-axial electrospray.

Sample Designation	Shell Materials	Core Materials
H-EAP	Hi-Cap^®^ 100, modified starch	Ethyl cellulose
		Retinyl palmitate
		Tween-20
H-EAPL	Hi-Cap^®^ 100 modified starch	Ethyl cellulose
		Retinyl palmitate
		Tween-20 Lecithin
H-EAPS	Hi-Cap^®^ 100 modified starch	Ethyl cellulose
		Retinyl palmitate
		Tween-20
		Soy protein acid hydrolysate
H-EAPC	Hi-Cap^®^ 100 modified starch	Ethyl cellulose
		Retinyl palmitate
		Tween-20
		Hy-case^®^ SF
H-EAPF	Hi-Cap^®^ 100 modified starch	Ethyl cellulose
		Retinyl palmitate
		Tween-20 Fibersol^®^–2
HF-EAP		Ethyl cellulose
	Hi-Cap^®^ 100 modified starch	Retinyl palmitate
	Fibersol^®^–2	Tween-20

## Data Availability

Data are contained within the article.
